# Supplementary material for: Dimethyl Itaconate Inhibits Melanogenesis in B16F10 Cells
Source: Antioxidants (Basel). 2023 Mar 10;12(3):692. doi: 10.3390/antiox12030692 (PMC10045371; doi:10.3390/antiox12030692)
Supplement: Supplementary file 1 [file antioxidants-12-00692-s001.zip › antioxidants-2171226-supplementary.pdf]

Experimental detail:

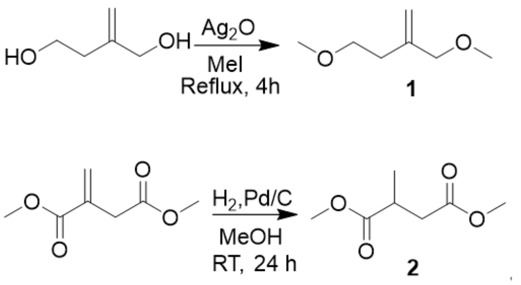

Supplementary Figure S1. Procedure for the synthesis of 4-methoxy-2-(methoxymethyl)but-1-ene (1): To a stirred solution of 2-methylenebutane-1,4-diol (0.010 g, 0.09 mmol) in 0.4 mL methyl iodide was added silver oxide (0.68 g, 0.29 mmol) and the mixture was refluxed for 4h. After completion, the reaction mixture was filtered through a pad of celite, the filtrate was evaporated and purified by column chromatography (DCM-Acetone, 9.5:0.5 v/v) to give **1** (0.004 g 31.5 %) as a colorless liquid.^{1}H NMR (400MHz, $CDCl_3$, δ): 5.07 (s, 1H), 4.98 (s, 1H), 3.88 (s, 1H), 3.52 (t, J = 6.8 Hz, 2H), 3.35 (s, 3H), 3.32 (s, 3H), 2.35 (t, J = 6.8 Hz, 2H). ^{13}C NMR (100 MHz, $CDCl_3$, δ): 113.18, 75.63, 71.13, 58.57, 57.85, 33.27. **Procedure for the synthesis of dimethyl 2-methylsuccinate (2):** To a stirred suspension of dimethyl 2-methylenesuccinate (0.040 g, 0.25 mmol) in methanol (2 mL) was added 10% Pd/C (0.006 g) and flask was sealed with a hydrogen balloon. The suspension was stirred for 24 h at room temperature. The resulting suspension was filtered through pad of celite and the filtrate was evaporated to give **2** (0.020 g, 49 %) as colorless liquid. ^{1}H NMR (400MHz, $CDCl_3$, δ): 3.70 (s, 3H), 3.68 (s, 3H), 2.98-2.89 (m, 1H), 2.75 (dd, J = 8.0, 16.0 Hz, 1H), 2.41 (dd, J = 6.0, 12.8 Hz, 1H), 1.22 (d, J = 7.2 Hz, 3H). ^{13}C NMR (100 MHz, $CDCl_3$, δ): 175.73, 172.31, 51.95, 51.74, 37.44, 35.73, 17.04.

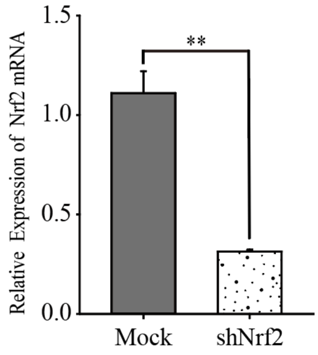

Supplementary Figure S2. The efficiency of Nrf2 shRNA in B16F10 cells. After Nrf2 was silenced by lentiviral transduction, the level of Nrf2 mRNAs in B16F10 cells was measured by real-time RT-PCR (n = 5).

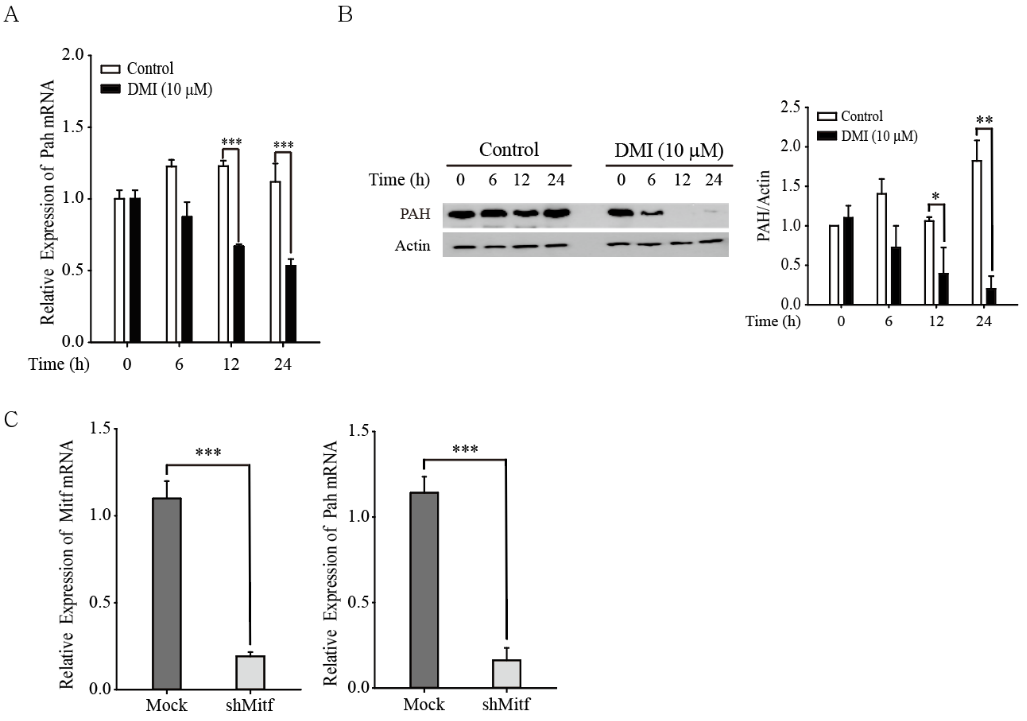

Supplementary Figure S3. DMI or knocking down Mitf inhibits phenylalanine hydroxylase (PAH) in B16F10 cells. (**A**) DMI inhibits the level of Pah mRNAs in B16F10 cells. B16F10 cells ($1.2x10^6$ cells) were exposed to DMI at various times and the level of Pah mRNA was measured by real-time RT-PCR analysis (n=5). (**B**) DMI inhibits the expression of PAH in B16F10 cells. B16F10 cells ($1.2x10^6$ cells) were exposed to DMI at various times and the protein expression of PAH was measured by Western blot analysis. The films of Western blot analysis were analyzed by densitometry and the amount of signals was calculated (n=3). (**C**) Knocking down Mitf inhibits the level of Mitf (Left Panel) and Pah (Right Panel) mRNAs in B16F10 cells. After Mitf was silenced by lentiviral transduction, the level of Mitf and Pag mRNAs in B16F10 cells was measured by real-time RT-PCR (n = 5).

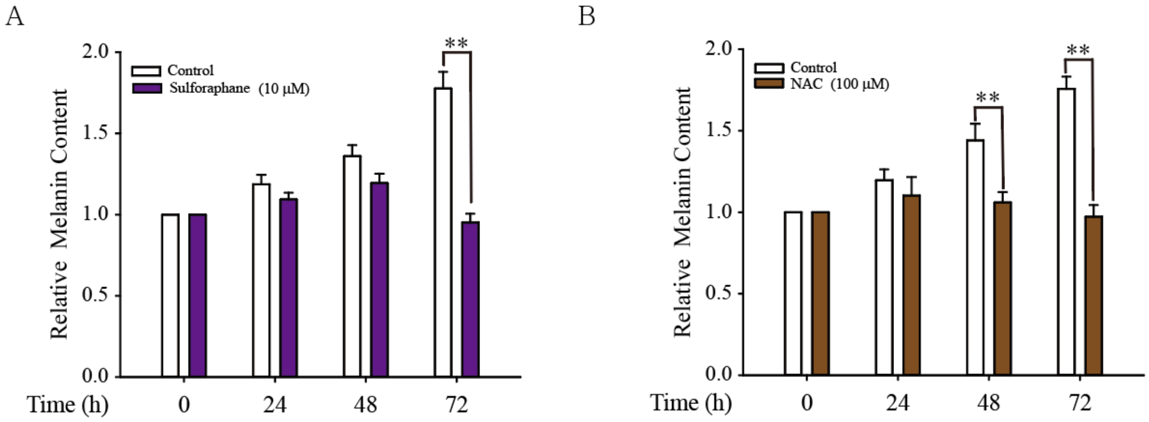

Supplementary Figure S4. Sulforaphane and N-acetylcysteine (NAC) inhibit the production of melanin in B16F10 cells. (**A**) sulforaphane inhibits the production of melanin in B16F10 cells. B16F10 cells (1×10^5 cells) were exposed to sulforaphane at various times and the amount of melanin was measured (n=3). (**B**) NAC inhibits the production of melanin in B16F10 cells. B16F10 cells (1×10^5 cells) were exposed to NAC at various times and the amount of melanin was measured (n=3).
